# Dr. E. Cutter

**Published:** 1879-02

**Authors:** 


### EDITORIAL, ETC.

---

The many friends whom Dr. E. Cutter made whilst in Washington and Baltimore last autumn, will be pleased to learn that he has been "winning golden opinions" in New York City lately, where he appeared before the New York Academy of Sciences, and lectured to a crowded house. The news correspondent of the *Boston Congregationalist*, writes as follows of the Doctor:

"The New York Academy of Sciences, on Monday evening, gave themselves and invited friends one of the richest scientific treats lately enjoyed here. The academy is our highest scientific body, numbering among its members the most eminent scientists and microscopists in the city. On this occasion, however, they borrowed from Boston your Dr. Ephraim Cutter, with the famous Tolles' one seventy-fifth inch objective lens, on the history, construction and uses of which he read a paper, with sciopticon illustrations. This is the lens to the work of which Joseph Cook has given such prominence, in his statements of the facts of biology, and by means of which he has been enabled to make large advances upon the information given by the best European authorities, on the intimate structure of the tissues of the body. The audience, large for the academy, were deeply interested in Dr. Cutter's account of the instrument and the remarkable illustrations, showing the vast enlargement of the minute objects to the examination of which it has been applied. The white corpuscles of the blood, *e. g.*, scarcely 1-1800 of an inch in diameter, were shown upon the screen, magnified to the size of two feet. The power of the lens, without amplifier, is not far from 7.500, or 56,250,000 times! Remarks upon the paper followed, in which the work of the lens was criticised as somewhat lacking in accuracy of definition and not superseding, if it even surpassed in practical value, the work of lower powers; but on the whole the new instrument met with a very cordial appreciation. In particular, Dr. Elsberg, one of our highest authorities, expressed his great admiration of such a piece of work. A lens whose largest face is only one-sixty-fourth of an inch in diameter, consisting of several pieces, which require to be ground and adjusted to each other with the utmost possible accuracy, and with a working distance of only 1-250 of an inch, he deemed such a triumph of mechanical skill as the world does not often see. The Academy warmly applauded Dr. Elsberg's commendation, and passed a hearty vote of thanks to Dr. Cutter."

We add, that Dr. Cutter received a few days ago an invitation from the President, the Earl of Shaftsbury, and the Council of the Victoria Philosophical Society of England, to become a member of that distinguished body. H.

